# Sarcobesity, but not visceral fat, is an independent risk factor for complications after radical resection of colorectal cancer

**DOI:** 10.3389/fnut.2023.1126127

**Published:** 2023-05-16

**Authors:** Zhewen Feng, Kai Pang, Mingwei Tian, Xiaozhe Gu, Huajun Lin, Xiaobao Yang, Yingchi Yang, Zhongtao Zhang

**Affiliations:** Department of General Surgery, Beijing Key Laboratory of Cancer Invasion and Metastasis Research and National Clinical Research Center for Digestive Diseases, Beijing Friendship Hospital, Capital Medical University, Beijing, China

**Keywords:** colorectal cancer, sarcobesity, sarcobesity index, visceral fat area, postoperative complications

## Abstract

**Background:**

The influence of body composition on the outcome of colorectal cancer surgery is controversial. The aim of this study was to evaluate the effects of visceral obesity and sarcobesity on the incidence of total and surgical complications after radical resection of colorectal cancer.

**Methods:**

We collected a total of 426 patients who underwent elective radical resection of colorectal cancer at Beijing Friendship Hospital, Capital Medical University from January 2017 to May 2018. According to the inclusion and exclusion criteria, 387 patients were finally included. A CT scan at the level of the L3-L4 intervertebral disk was selected to measure the values of visceral fat area and skeletal muscle area. Multivariate analysis was used to explore the independent risk/protective factors affecting postoperative complications.

**Results:**

128 (33.1%) patients developed complications, and 44 (11.4%) patients developed major complications. Among them, 111 patients developed surgical complications and 21 developed medical complications. Visceral fat area (*Z* = −3.271, *p* = 0.001), total fat area (Z = −2.613, *p* = 0.009), visceral fat area to subcutaneous fat area ratio (V/S, Z = −2.633, *p* = 0.008), and sarcobesity index (*Z* = −2.282, *p* = 0.023) were significantly associated with total complications. Visceral fat area (*Z* = −2.119, *p* = 0.034) and V/S (*Z* = −2.010, *p* = 0.044) were significantly associated with total surgical complications. Sarcobesity index, smoking, stoma, blood loss, surgery time, and American Society of Anesthesiology (ASA) score were selected as risk factors for total postoperative complications according to LASSO regression. Multivariate logistic regression analysis suggested that sarcobesity index was an independent risk factor for postoperative total complications and surgical complications. Subgroup analysis suggested that albumin level was an independent protective factor for postoperative total complications in male patients. Smoking, operative time, and sarcobesity index were independent risk factors, and cholesterol was an independent protective factor for total postoperative complications in female patients.

**Conclusion:**

Increased sarcobesity index is an independent risk factor for postoperative complications in patients with colorectal cancer, while visceral fat area is not. For female patients, smoking, operation time, and obesity index are independent risk factors for postoperative complications, while cholesterol is an independent protective factor. For male patients, serum albumin is an independent protective factor for postoperative complications.

## Introduction

Overweight and obesity are important global health issues with an important role in the development and prognosis of several cancers (1). Obesity increases the difficulty of surgery, incurs high costs, and complicates the surgical treatment of colorectal cancer (2, 3). Body mass index (BMI) is commonly used to determine obesity. However, BMI as a risk profiler for early postoperative outcomes has been questioned (4–6). The association between obesity as diagnosed by BMI and adverse outcomes after colorectal cancer surgery remains controversial (7, 8). These conflicting data may be attributed to the inability of BMI to assess the proportions of fat and lean tissue (9).

The distribution of adipose tissue in the body is diverse, and different fat depots have different metabolic activities. Visceral fat is considered a more accurate parameter than subcutaneous fat to reflect dysfunctional adipose tissue, which is a major cause of various obesity-related comorbidities (10). Visceral fat is metabolically active, which can lead to a chronic inflammatory state and increase the risk of insulin resistance and metabolic syndrome (11, 12). Recent studies used computed tomography (CT) imaging to identify and quantify visceral and subcutaneous fat, which is more accurate than BMI and waist circumference (WC) (13–15). Some studies showed that visceral fat area (VFA) ≥ 100 cm^2^ is associated with metabolic syndrome and is a risk factor for poor prognosis and prolonged hospital stay after colorectal surgery (16).

Another concept that needs attention is sarcobesity (SO). Sarcopenia, which can occur independently of adiposity, is associated with physical disability, injuries, and mortality in individuals with non-malignant disease (17, 18). SO, the simultaneous occurrence of visceral obesity and low muscle mass, represents a worst-case scenario because it combines the health risks of visceral obesity and depleted lean mass (19). Pedrazzani et al. (20) reported that SO is a risk factor for developing cardiac complications and prolonged postoperative ileus (PPOI) after laparoscopic resection for colorectal cancer (CRC). The aim of this study was to evaluate the effects of VFA and SO on the incidence of total and surgical complications after radical resection of CRC.

## Materials and methods

In this retrospective cohort study, we collected a total of 426 patients who underwent elective radical resection of CRC at Beijing Friendship Hospital, Capital Medical University from January 2017 to May 2018. All procedures were performed by experienced surgeons and their teams. The study protocol was approved by the Ethics committee of Beijing Friendship Hospital, Capital Medical University, with the number “2022-P2-104-01.”

Inclusion criteria included: (1) age > 18 years; (2) an abdominal CT scan within 30 days before surgery; (3) only elective radical resection of colorectal cancer; (4) complete clinical data; and (5) complete postoperative pathological data of colorectal cancer. Exclusion criteria included: (1) absence or inability to obtain an abdominal CT scan within 30 days before surgery; (2) combined with other organ resection; (3) incomplete clinical data; (4) palliative surgery or emergency surgery; and (5) previous history of other malignancies. Patients without available or detailed CT images (*n* = 32) were excluded. Patients who underwent combined evisceration (*n* = 4) were excluded. One patient had a history of breast cancer, one had a history of thyroid cancer, and one had a history of ovarian cancer. Finally, 387 patients were included in our retrospective study.

### Data collection

The following data were collected and analyzed retrospectively: (1) clinical data, including age, sex, smoking history, diabetes mellitus (DM), Charlson comorbidity index (CCI) (21), American Society of Anesthesiology (ASA) score, history of abdominal operation, neoadjuvant therapy, cancer (colon cancer or rectal cancer), operation (open, laparoscopic or convert to open), stoma, hemoglobin (HGB), albumin (ALB), triglyceride (TG), high-density lipoprotein cholesterol (HDL-C), fasting glucose, cholesterol, carcinoembryonic antigen (CEA), carbohydrate antigen199 (CA199), operative time, vascular invasion, and TNM classification; (2) postoperative complications: postoperative complications were graded according to the Clavien-Dindo Classification (CDC) (22) and divided into surgical complications (SC) and medical complications (MC). Surgical complications included anastomotic leakage (AL), wound infection, bleeding, abdominal infection, bladder dysfunction, intestinal obstruction, and rectovaginal fistula. Medical complications included cardiologic complications, respiratory complications, sepsis, and urinary tract infection (UTI). CDC ≥ III was defined as major complications (23).

### CT-based quantification of body composition

In this study, all patients underwent abdominal CT scans within 30 days before surgery at Beijing Friendship Hospital, Capital Medical University. A scan at the level of the L3-L4 intervertebral disk was selected to measure the values of visceral fat area (VFA) and subcutaneous fat area (SFA) (14). VFA and SFA were identified and quantified using Hounsfield units (HU) thresholds of −150 to −50 HU and − 190 to −30 HU, respectively (24). We defined VFA as the intra-abdominal adipose tissue area within the parietal peritoneum, excluding the paraspinal muscle, intervertebral bodies, and intramuscular fat. SFA was defined as the adipose tissue external to the peritoneum and back muscle (25). Total fat area (TFA) was defined as the sum of VFA and SFA. Visceral fat area to subcutaneous fat area ratio (V/S) was also calculated to assess the degree of visceral obesity. Skeletal muscle was identified and quantified by use of HU thresholds (−29 to +150) (19). Skeletal muscle area (SMA) contains psoas, paraspinal muscles (erector spinae, quadratus lumborum), and abdominal wall muscles (transversus abdominus, external and internal obliques, and rectus abdominus). SO was defined using the VFA/SMA ratio (Sarcobesity Index). Because the standard cutoff values for VFA and SO were not uniform, we analyzed them as continuous variables.

**Figure 1:**
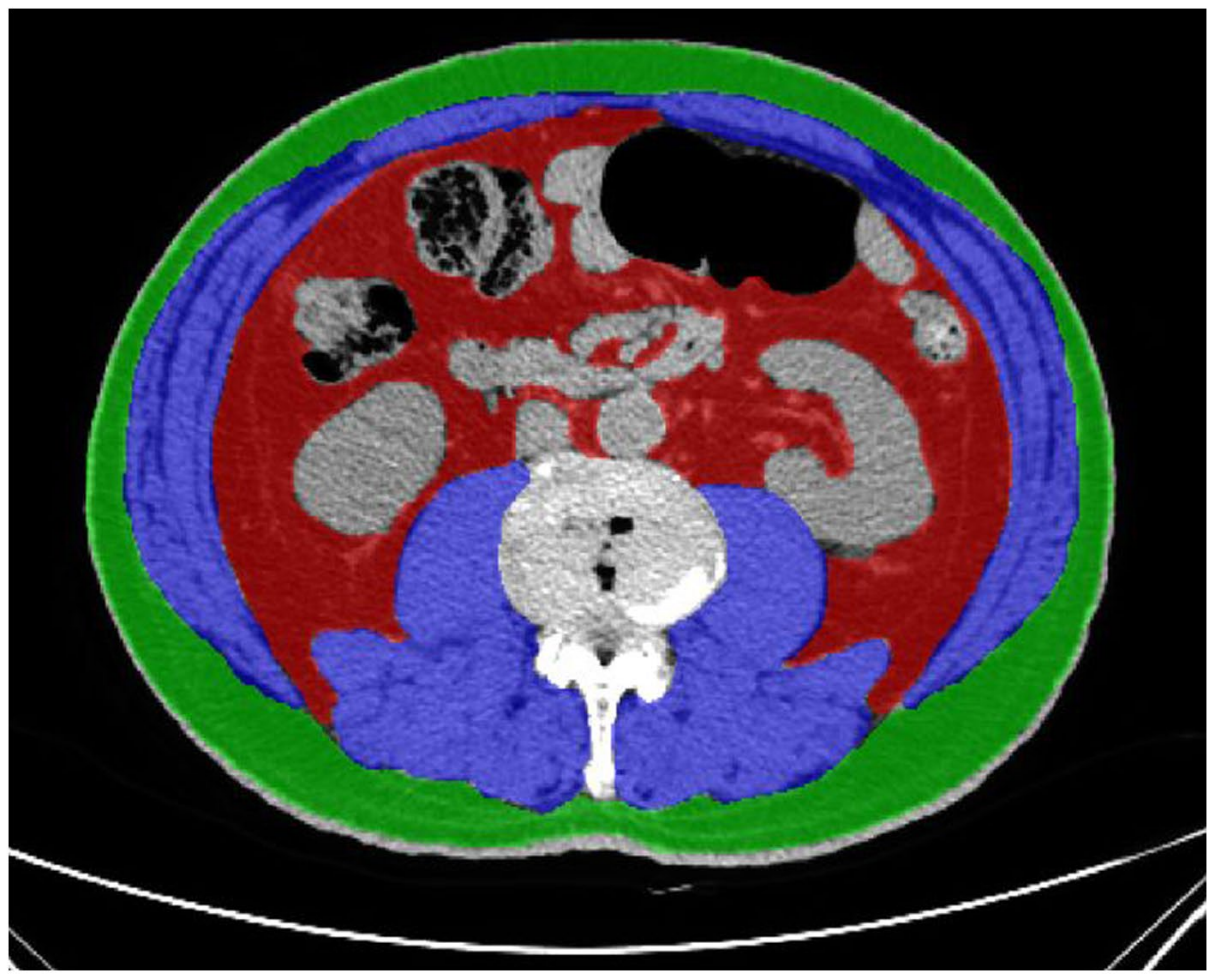
Cross sectional CT imaging at L3-L4 intervertebral disk. Muscle tissue is shown in blue color, subcutaneous fat tissue in green color, and visceral fat tissue in red color. Visceral fat tissue and subcutaneous fat tissue were identified and quantified using Hounsfield units (HU) thresholds of −150 to −50 HU and − 190 to −30 HU. Skeletal muscle was identified and quantified by use of HU thresholds (−29 to +150).

Fat and skeletal muscle areas were labeled and measured semiautomatically by two experienced radiologists using ITK-SNAP version 3.8.0 (Figure 1).

### Statistical analysis

We describe categorical variables as numbers with percentages. Continuous variables were described as means with SDs or medians with interquartile ranges, depending on the data distribution. VFA, SFA, TFA, V/S, and sarcobesity index were treated as continuous variables. Clinical variables were compared using independent samples *t* test, Pearson’s chi-square test or Mann–Whitney U test as appropriate. Two continuous variables were evaluated using Spearman’s rank correlation coefficient. LASSO regression was used to select the variables affecting the occurrence of postoperative complications. Multivariate analysis was performed for the selected variables with multiple logistic regression analysis. To increase the interpretability of the results, subgroup analysis was performed according to sex. In the subgroup analysis, LASSO regression and multiple logistic regression analysis were performed for male/female individually for variable selection and multivariate analysis. A receiver operating characteristic (ROC) curve analysis was used to develop a cut-off for sarcobesity index associated with postoperative outcome in males/females. And the cut-off values for sarcobesity index that maximized the Youden index (sensitivity + specificity −1) were defined as optimal. The patients were divided into control group and SO group according to the cut-off value of sarcobesity index. All of the tests were two-sided and considered statistically significant at *p* < 0.05. SPSS version 26.0 and R version 4.1.0 were used for statistical analysis. “glmnet” package was used to perform the LASSO regression. SPSS was used to perform multiple logistic regression analysis and ROC curves.

## Results

### Patient characteristics

387 adult patients underwent elective radical resection of CRC and met inclusion and exclusion criteria. Demographics and operative characteristics were shown in Table 1. The median age was 64 years, median BMI was 23.18 kg/m^2^, 246 (63.6%) patients were female, and 141 (36.4%) patients were male. There were 247 (63.8%) patients with colon cancer and 140 (36.2%) patients with rectal cancer. 104 (26.9%) patients underwent open surgery, 255 (65.9%) patients underwent laparoscopic surgery, and 28 (7.2%) patients underwent laparoscopic conversion to open surgery.

**Table 1:** Patients’ characteristics.

Characteristics	Number of cases (*n* = 387)
Age (years)	64 (58,72)
Sex	
Female	246 (63.6)
Male	141 (36.4)
BMI (kg/m^2^)	23.18 (21.3,25.69)
Smoking	142 (36.7)
Diabetes mellitus	81 (20.9)
CCI	
≤2	356 (92.0)
>2	31 (8.0)
Abdominal surgery	98 (25.3)
Neoadjuvant	40 (10.3)
ASA score	
<III	208 (53.7)
≥III	179 (46.3)
Cancer	
Colon cancer	247 (63.8)
Rectal cancer	140 (36.2)
TNM stage	
<III	229 (59.2)
≥III	158 (40.8)
Surgery	
Open	104 (26.9)
Laparoscope	255 (65.9)
Convert to open	28 (7.2)
Stoma	111 (28.7)
Blood loss (mL)	50 (50,100)
Operative time (min)	230 (170,290)
Lymph node	15 (11,20)
Vascular invasion	157 (40.6)
TG (mmol/L)	1.19 (0.89,1.52)
HDL-C (mmol/L)	1.01 (0.84,1.21)
Glucose (mmol/L)	5.15 (4.74,5.74)
Cholesterol (mmol/L)	4.29 (3.73,4.94)
CEA (ng/mL)	3.14 (1.72,8.91)
CA199 (kU/L)	10.85 (5.40,22.50)
HGB (g/L)	122 (103,135)
Albumin (g/L)	36.9 (33.9,39.3)

The measurement data were expressed by median (interquartile ranges), and the enumeration data were expressed by the number of cases (percentage). CCI, Charlson comorbidity index; ASA score, American Society of Anesthesiology classification system score; TG, triglyceride; HDL-C, high-density lipoprotein cholesterol; CEA, carcinoembryonic antigen; CA199, carbohydrate antigen199; and HGB, hemoglobin.

### Association between body composition and postoperative complications

128 (33.1%) patients developed complications, and 44 (11.4%) patients developed major complications (Table 2). Among them, 111 patients developed SC and 21 developed medical complications. The incidence of postoperative total complications was significantly associated with VFA, TFA, V/S, and sarcobesity index. Among them, surgical complications was significantly associated with VFA and V/S, but anastomotic leakage was only significantly associated with V/S. For medical complications and major complications, the differences in VFA, SFA, TFA, V/S, and SO were statistically significant.

**Table 2:** The correlation of sarcobesity index, visceral fat area (VFA), subcutaneous fat area (SFA), total fat area (TFA), ratio of visceral fat area to subcutaneous fat area (V/S), and postoperative complications.

		VFA		SFA		TFA		V/S		Sarcobesity index	
	*n*	t/Z	*p*	t/Z	*p*	t/Z	*p*	t/Z	*p*	t/Z	*p*
Total complication	128	-3.271	0.001	−0.757	0.449	−2.613	0.009	−2.633	0.008	−2.282	0.023
Surgical complications	111	−2.119	0.034	−0.136	0.892	−1.538	0.124	−2.01	0.044	−0.969	0.332
Anastomotic leakage	26	−1.307	0.191	−0.983	0.326	−0.673	0.501	−2.21	0.027	−0.804	0.421
Bleeding	20	−0.256	0.798	−1.123	0.262	−0.857	0.391	−0.139	0.89	−0.049	0.961
Abdominal infection	35	−1.994	0.046	−1.299	0.194	−2.084	0.037	−1.687	0.092	−1.587	0.113
Wound infection	15	−0.364	0.716	−0.82	0.412	−0.73	0.465	−0.234	0.815	−0.364	0.716
Intestinal obstruction	24	−1.777	0.076	−0.688	0.492	−0.91	0.363	−2.708	0.007	−1.513	0.13
Bladder dysfunction	7	−0.474	0.636	−0.91	0.363	−0.743	0.457	−0.218	0.827	−0.658	0.51
Rectovaginal fistula	1	−1.41	0.159	−0.103	0.918	−1.025	0.305	−1.589	0.112	−1.705	0.088
Medical complications	21	−3.734	<0.001	−1.397	0.162	−3.246	0.001	−2.738	0.006	−4.068	<0.001
Cardiologic complications	9	−2.406	0.016	−1.595	0.111	−2.408	0.016	−1.119	0.263	−2.457	0.014
Respiratory complications	7	−1.91	0.056	−0.984	0.325	−1.742	0.081	−1.156	0.248	−2.576	0.01
UTI	1	−1.41	0.159	−0.103	0.918	−1.025	0.305	−1.589	0.112	−1.705	0.088
Acute kidney injury	1	−1.41	0.159	−0.103	0.918	−1.025	0.305	−1.589	0.112	−1.705	0.088
Sepsis	3	−2.453	0.014	−1.062	0.288	−2.161	0.031	−2.166	0.03	−1.407	0.159
Major complication	44	−3.406	0.001	−0.276	0.783	−2.235	0.025	−3.473	0.001	−2.951	0.003

UTI, urinary tract infection; VFA, visceral fat area; SFA, subcutaneous fat area; TFA, total fat area; and V/S, visceral fat area to subcutaneous fat area ratio.

### Variables selection and multivariate analysis

**Figure 2:**
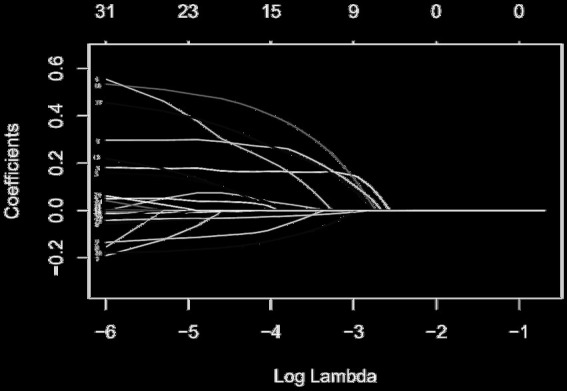
LASSO coefficient profiles of the 31 risk factors affecting the occurrence of postoperative total complications in patients with colorectal cancer. The risk factors corresponding to the number of each curve are shown in Supplementary Table 2.

**Figure 3:**
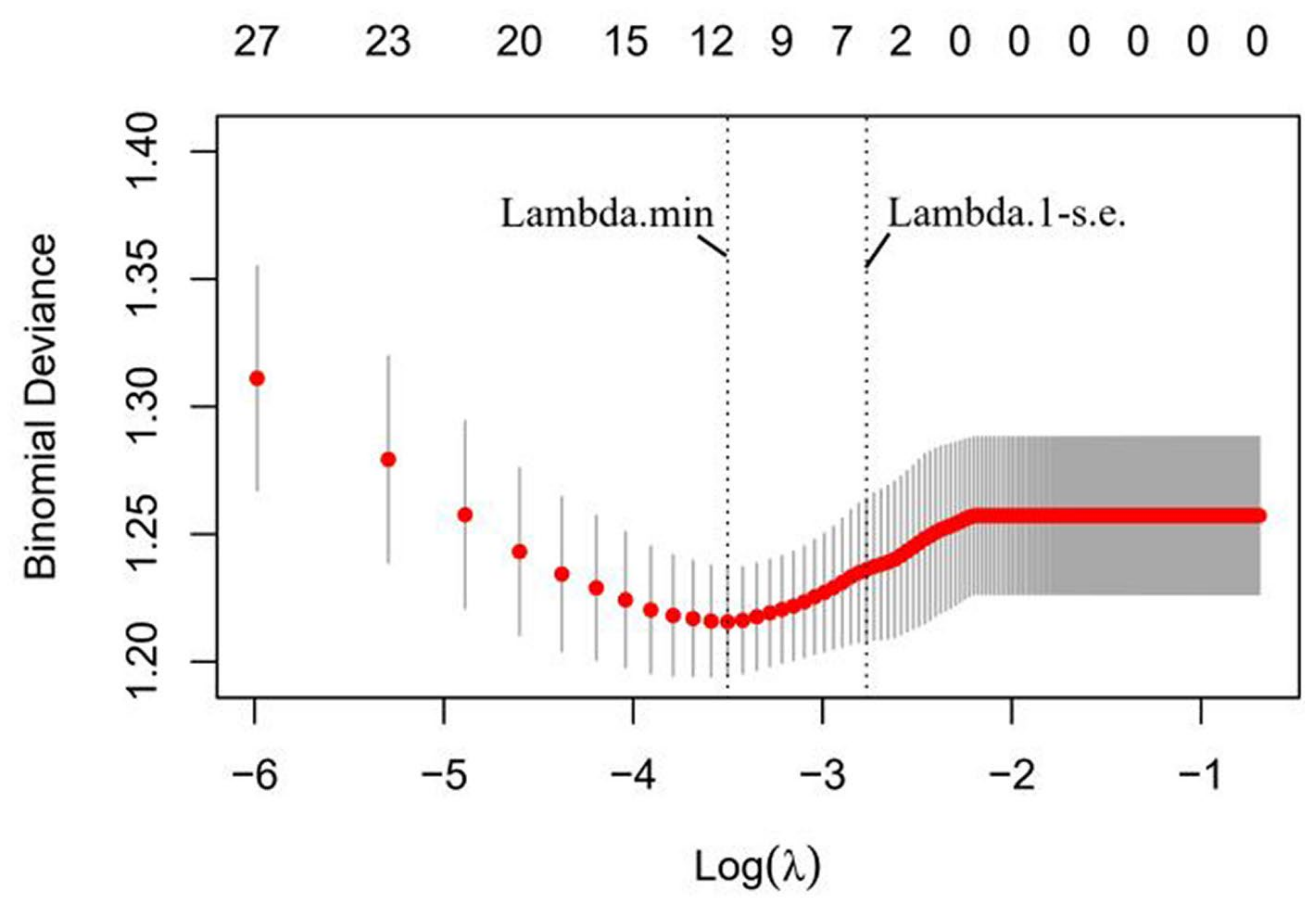
Six risk factors selected using LASSO Cox regression analysis. The two dotted vertical lines were drawn at the optimal scores by minimum criteria and 1-SE criteria (at 1-SE criteria including sarcobesity index, smoking, stoma, blood loss, surgery time, and ASA ≥ III).

To identify independent risk factors for total complications and surgical complications among the different variables measured, all available clinical indicators, including clinicopathological features, were subjected to LASSO regression (Figure 2). Further disciplinary regression was performed to take sarcobesity index, smoking, stoma, blood loss, operative time, and ASA as factors for postoperative total complications (Figure 3). Multiple Logistic regression analysis was then performed and showed that smoking, ASA ≥ III, increased sarcobesity index, and operative time were independent risk factors for postoperative total complications (Table 3). Same procedure was used to screen for independent risk factors for surgical complications (Table 4). The results showed that sarcobesity index, smoking, operative time were independent risk factors, and cholesterol was an independent protective factor for surgical complications. Smoking, increased sarcobesity index and operative time, and decreased cholesterol levels were associated with increased risk of surgical complications.

**Table 3:** Multivariate analysis between total complications and risk/protective factors.

Parameters	B	SE	Wald	OR (95%CI)	*p*
Sarcobesity index	0.512	0.209	5.986	1.669 (1.107,2.515)	0.014
Smoking	0.589	0.237	6.165	1.803 (1.132,2.871)	0.013
Stoma	0.469	0.272	2.971	1.599 (0.938,2.726)	0.085
Blood loss	0.002	0.001	3.794	1.002 (1.000,1.004)	0.051
Operative time	0.003	0.002	4.222	1.003 (1.000,1.006)	0.040
ASA ≥ III	0.539	0.235	5.243	1.715 (1.081,2.720)	0.022

The parameters involved in the multivariate analysis were screened by LASSO regression. ASA, American Society of Anesthesiology classification system.

**Table 4:** Multivariate analysis between surgical complications and risk/protective factors.

Parameters	B	SE	Wald	OR (95%CI)	*p*
Sarcobesity index	0.436	0.209	4.372	1.547 (1.028,2.328)	0.037
Smoking	0.754	0.245	9.482	2.125 (1.315,3.432)	0.002
Blood loss	0.001	0.001	1.204	1.001 (0.999,1.002)	0.273
Operative time	0.004	0.001	9.265	1.004 (1.002,1.007)	0.002
Cholesterol	−0.381	0.14	7.438	0.683 (0.519,0.898)	0.006

The parameters involved in the multivariate analysis were screened by LASSO regression.

**Figure 4:**
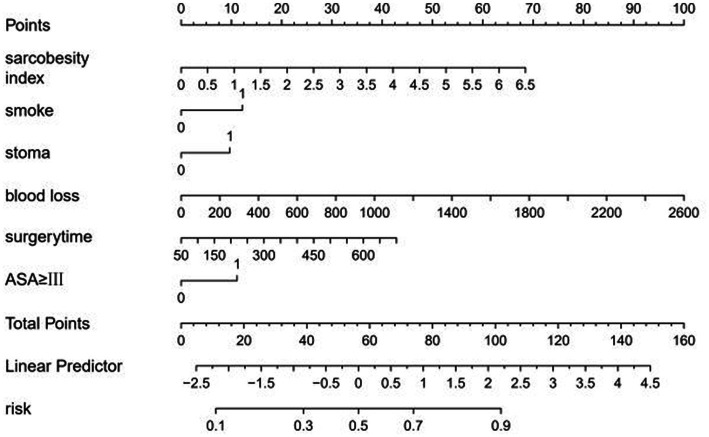
Nomogram including sarcobesity index (SO), smoking, stoma, blood loss, surgery time, and ASA ≥ III for total complications after radical resection of colorectal cancer.

**Figure 5:**
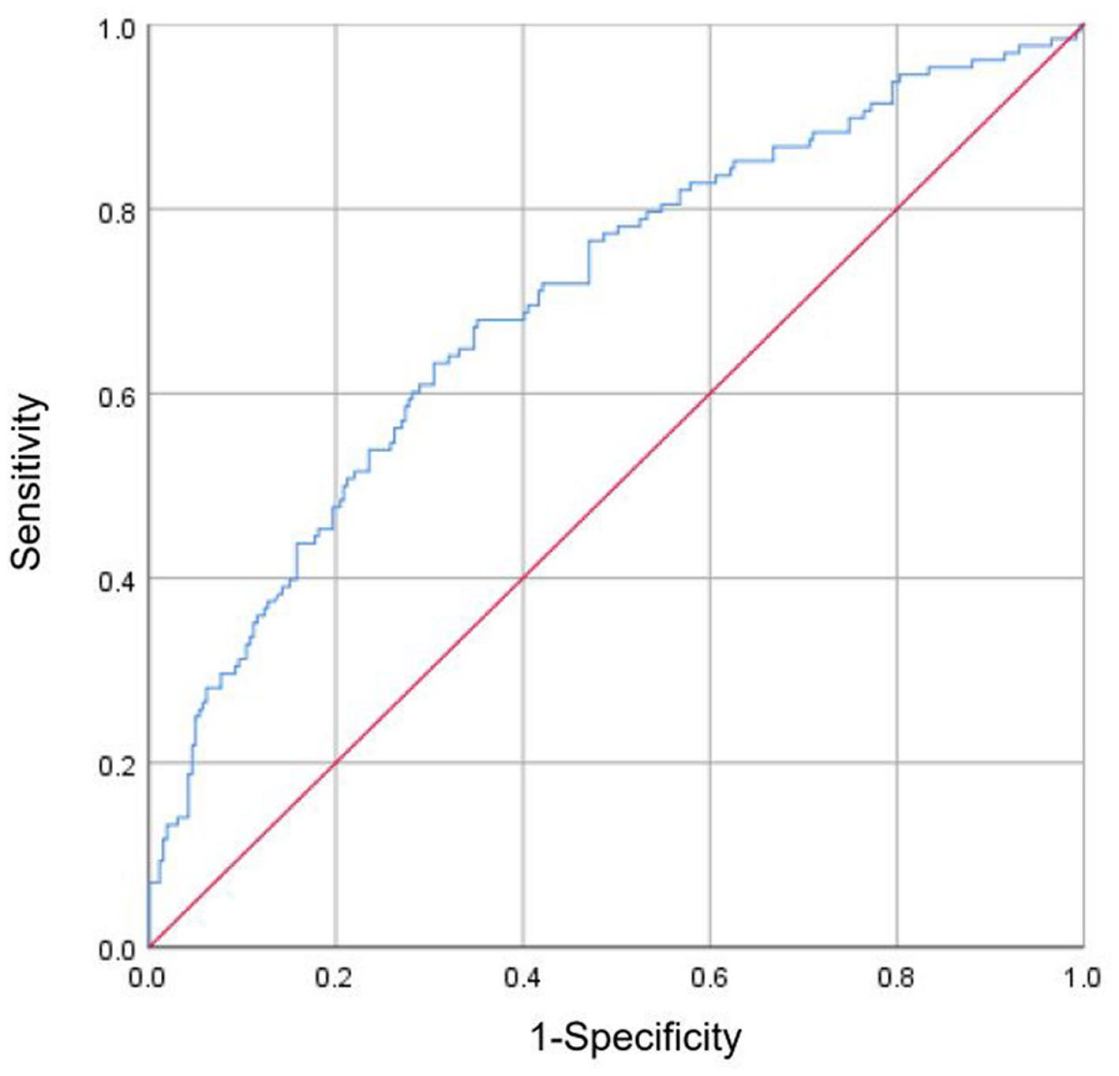
Receiver operating characteristic (ROC) analysis was performed to determine the reliability of the nomogram model for predicting the occurrence of total complications after radical resection of colorectal cancer. Area under the ROC curve (AUC) was 0.702.

Nomogram was constructed to predict total complications based on factors determined by multiple logistic regression analysis (Figure 4). ROC analysis was performed to determine the accuracy of logistic regression model. Area under the ROC curve (AUC) was 0.702 (Figure 5).

### Variables selection and multivariate analysis of male patients for subgroup analysis

**Figure 6:**
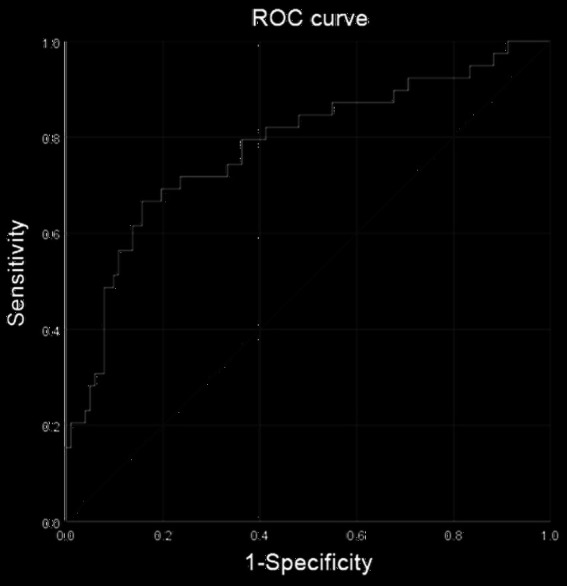
Receiver operating characteristic (ROC) analysis was performed to determine the reliability of the nomogram model for predicting the occurrence of total complications after radical resection of colorectal cancer in male patients. The area under the ROC curve (AUC) was 0.779.

Because body composition was clearly different between males and females, subgroup analyses were performed according to sex. A total of 141 male patients were included in this study. All available clinical measures, including clinicopathological features and all obesity-related measures, were subjected to LASSO regression (Supplementary Figure 1; Figure 2). Finally, sarcobesity index, albumin, ASA score, stoma, and blood loss were selected as the factors of postoperative total complications, and then these factors were analyzed by multiple Logistic regression analysis. Multivariate analysis showed that albumin level was an independent protective factor for postoperative total complications in male patients (Table 5). The decrease of albumin increased the risk of postoperative total complications in male patients. ROC analysis was used to determine the accuracy of the logistic regression model. The AUC was 0.779 (Figure 6). A cutoff value of sarcobesity index≥1.395 cm^2^, selected for maximizing Youden’s J statistics, was associated with 46.2% sensitivity and 75.5% specificity.

**Table 5:** Multivariate analysis between total complications and risk/protective factors of male patients.

Parameters	B	SE	Wald	OR (95%CI)	*p*
Sarcobesity index	0.430	0.39	1.215	1.54 (0.72,3.30)	0.27
Albumin	−0.148	0.054	7.426	0.86 (0.78,0.96)	0.006
ASA ≥ III	0.798	0.477	2.791	2.22 (0.87,5.66)	0.095
Stoma	0.540	0.484	1.247	1.72 (0.67,4.43)	0.264
Blood loss (mL)	0.003	0.002	1.073	1.00 (1.00,1.01)	0.300

The parameters involved in the multivariate analysis were screened by LASSO regression. ASA, American Society of Anesthesiology classification system.

### Variables selection and multivariate analysis of female patients for subgroup analysis

**Figure 7:**
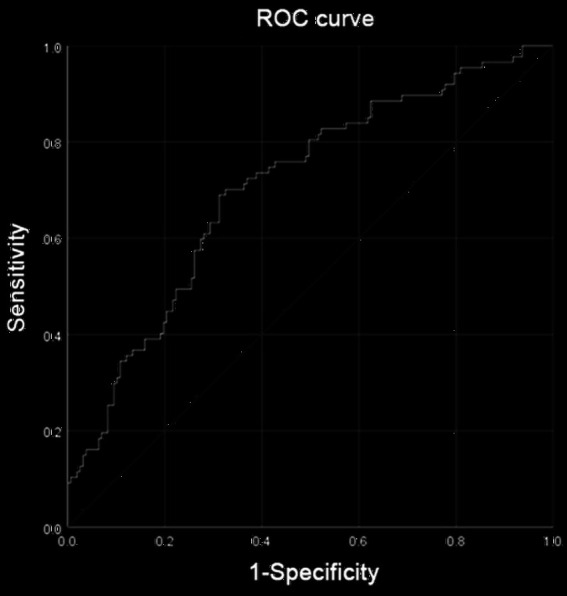
Receiver operating characteristic (ROC) analysis was performed to determine the reliability of the nomogram model for predicting the occurrence of total complications after radical resection of colorectal cancer in female patients. The area under the ROC curve (AUC) was 0.707.

A total of 246 female patients were included in this study. Smoking, operative time, cholesterol, and sarcobesity index were selected as the factors of postoperative total complications in female patients after LASSO regression (Supplementary Figure 3; Figure 4). Multiple Logistic regression analysis showed that smoking, operative time, and sarcobesity index were independent risk factors, and cholesterol was an independent protective factor for total postoperative complications in female patients (Table 6). Smoking, increased operative time and muscle mass, and decreased cholesterol levels increased the risk of overall postoperative complications in female patients. ROC analysis was used to determine the accuracy of the logistic regression model. The AUC was 0.707 (Figure 7). In the case of maximum Youden’s J statistic, the cut-off value of muscle adiposity index was ≥0.845 cm^2^, with a sensitivity of 71.9% and a specificity of 42%.

**Table 6:** Multivariate analysis between total complications and risk/protective factors of female patients.

Parameters	B	SE	Wald	OR (95%CI)	*p*
Smoking	0.703	0.293	5.745	2.02(1.14,3.59)	0.017
Operative time	0.006	0.002	13.973	1.01(1.00,1.01)	<0.001
Cholesterol	−0.361	0.170	4.520	0.70(0.50,0.97)	0.033
Sarcobesity index	0.607	0.273	4.953	1.84(1.08,3.13)	0.026

The parameters involved in the multivariate analysis were screened by LASSO regression.

### Comparison of clinical characteristics between control group and SO group

Sarcobesity was defined as sarcobesity index ≥1.395 cm^2^ in male patients and ≥ 0.845 cm^2^ in female patients. The patients were divided into SO group and control group. The differences in clinical characteristics between the control and SO groups were shown in Table 7. Patients had a higher age [66 (59,74) vs. 62 (55,70) years, *Z* = −3.642, *p* < 0.001], more diabetes mellitus (30.3 vs. 11.1%, χ^2^ = 21.521, *p* < 0.001), more stoma (33.3 vs. 23.8%, χ^2^ = 4.288, *p* = 0.038), less harvested lymph nodes [14 (10, 19) vs. 16 (12, 22), *Z* = −3.548, *p* < 0.001], more blood loss [50 (50,100) vs. 50(50,150), *Z* = −2.428, *p* = 0.015] and more vascular invasion (46.4 vs. 34.9%, χ^2^ = 5.276, *p* = 0.022) in the SO group. However, there was no significant difference in smoking, CCI, cancer, neoadjuvant, TNM stage, operative time, and surgical methods between the two groups (*p* > 0.05). Multiple tests were used to correct for statistical differences among the three surgical procedures, and the results suggested that there was no statistically significant difference in surgical approach between the control and SO groups (Supplementary Table 1).

**Table 7:** Differences in patient characteristics and postoperative pathological outcomes between sarcobesity group (SO) and control group.

	Control group (*n* = 189)	SO group (*n* = 198)	Z/χ^2^	*p*
Age	62(55,70)	66(59,74)	−3.642	<0.001
Sex			37.916	<0.001
Female	91(48.1%)	155(78.3%)		
Male	98(51.9%)	43(21.7%)		
Smoking	62(32.8%)	80(40.4%)	2.404	0.121
DM	21(11.1%)	60(30.3%)	21.521	<0.001
CCI			2.405	0.121
≤2	178(94.2%)	178(89.9%)		
>2	11(5.8%)	20(10.1%)		
Cancer			0.119	0.730
Colon cancer	119(63.0%)	128(64.6%)		
Rectal cancer	70(37.0%)	70(35.4%)		
Neoadjuvant	17(9.0%)	23(11.6%)	0.717	0.397
TNM stage			3.594	0.058
<III	121(64.0%)	108(54.5%)		
≥III	68(36.0%)	90(45.5%)		
Stoma	45(23.8%)	66(33.3%)	4.288	0.038
Lymph nodes	16(12,22)	14(10,19)	−3.548	<0.001
Blood loss (mL)	50(50,100)	50(50,150)	−2.428	0.015
Operative time (min)	220(160,290)	240(180,290)	−1.605	0.108
Vascular invasion	66(34.9%)	91(46.4%)	5.276	0.022
Surgery^a^			5.282	0.071
Open	50(26.5%)	54(27.3%)		
Laparoscope	131(69.3%)	124(62.6%)		
Convert to open	8(4.2%)	20(10.1%)		

The measurement data were expressed by median (interquartile ranges), and the enumeration data were expressed by the number of cases (percentage). SO was defined as sarcobesity index ≥ 1.395 cm^2^ in male patients and ≥ 0.845 cm^2^ in female patients. DM, diabetes mellitus; CCI, Charlson comorbidity index.^a^Multiple testing correction was used, as detailed in Supplementary Table 1.

## Discussion

This retrospective cohort study revealed that increased sarcobesity index is an independent risk factor for postoperative total complications in female patients, while visceral fat was not an independent risk factor for postoperative total complications in either male or female patients. Our findings suggest that sarcobesity, the simultaneous occurrence of visceral obesity and low muscle mass, could be a better predictor of postoperative complications in CRC than visceral obesity at least in females. Several studies have compared the associated of visceral obesity and sarcobesity with the outcome of CRC surgery (20, 26). However, all of these were studies of European patients, who have different prevalence of obesity, obesity criteria, and skeletal muscle content than Asian populations (27, 28). In our study, only 10 patients (2.6%) met the criteria for obesity (BMI ≥ 30 kg/m^2^), compared with 20–40% of the European population (29). In addition, these studies have a drawback of assessing sarcobesity index as a dichotomous variable, which assumes a predetermined cutoff value of sarcobesity index to define sarcobesity, when in fact, no consensus cutoff value exists. It is worth mentioning that many studies have used different criteria to define visceral obesity for males and females, while few studies have developed separate criteria to define SO according to sex. This may be the first study to investigate the association of VFA and sarcobesity index with postoperative complications of CRC in an Asian population.

Our study found that VFA was significantly associated with total complications, surgical complications, medical complications, and major complications (*p* < 0.05), but was not an independent risk factor for total complications and SC. Other studies have reached different conclusions (5, 9, 15). Watanabe et al. (5) reported that visceral obesity (cutoff value of 100 cm^2^) independently predicted the incidence of overall postoperative complications of laparoscopic surgery for colon cancer (*p* = 0.007). This difference in results may be due to our inclusion of open surgery. Surgeons tend to prefer open surgery in patients with high visceral fat. Different cutoff values of VFA may be another reason for the difference (30, 31). Frostberg et al. (32) reported that VFA (cutoff value of 130 cm^2^) were unable to predict complications after CRC surgery, contrary to the results of Watanabe et al. (5). Furthermore, we abandoned multivariate analysis of major complications and anastomotic leakage, because the number of these complications was too small and could easily lead to bias. By ROC analysis, we obtained the best cutoff value of sarcobesity index. We found SO patients had a lower number of lymph node dissection and a higher rate of conversion to open surgery. The College of American Pathologists has established guidelines for the pathologic evaluation of colorectal cancer resection specimens and recommended that a minimum of 12 lymph nodes should be removed (33). In three studies, improved survival was observed with more than 12 lymph nodes evaluated (34–36). The first quartile of the number of dissected lymph nodes [14 (10,19)] in the SO group exceeded the cutoff value. This means that more than a quarter of the patients in the SO group were unable to meet the requirements. The difference of dissected lymph nodes can have two explanations: the lymph nodes are located deep in the perivascular fat, and the large amount of visceral fat may affect the adequate resection of the lymph nodes (37); adipose tissue adheres to the mesentery, making it difficult for pathologists to identify lymph nodes (38). In our study, the SO group had more stomas and more intraoperative blood loss, but the operative time was not statistically different from the control group. We venture to hypothesize that advances in surgical techniques and devices may have markedly reduced the effect of large amounts of visceral fat on procedural time. However, the large amount of visceral adipose tissue still significantly increases the amount of intraoperative blood loss and the rate of stoma. This may be because separating large amounts of adipose tissue and exposing vital structures inevitably causes more bleeding and makes the surgeon more inclined to choose a prophylactic stoma.

In our study, we perceived an interesting finding that the proportion of females was significantly higher than that of males in the SO group (78.3 vs. 21.7%, χ^2^ = 37.916, *p* < 0.001). However, in several studies based on European populations, sarcobesity group had a high proportion of males (19, 20, 26). The fact that these studies did not define SO separately for males and females may be one reason for this discrepancy. Besides, VFA and SMA, two parameters that make up the formula for sarcobesity index, have sex differences. In our study, Asian females seem to have relatively more skeletal muscle loss or visceral fat accumulation. This may be due to the fact that Asian populations have more visceral fat compared to European populations (39). On the other hand, relatively low intake of red meat and impaired skeletal muscle mass in postmenopausal females may also contribute to sarcobesity in elder females (40, 41). Sarcobesity is an independent risk factor for postoperative complications of colorectal cancer in female patients. Therefore, it is of great significance to pay attention to this group of elderly females and improve their skeletal muscle loss and visceral fat accumulation before surgery.

Multivariate analysis showed that serum albumin and cholesterol were independent protective factors for postoperative complications in male and female patients, respectively. Preoperative serum albumin is well-known as an effective predictor of the outcome of colorectal cancer surgery and a component of nutritional screenings, such as the Prognostic Nutritional Index and Nutritional Risk Index (42–44). Hypoalbuminemic patients (serum albumin <35 g/L) are reported to have significantly higher rates of postoperative morbidity and mortality, as well as complications related to wounds and anastomosis compared with patients with normal serum albumin levels (45). For serum cholesterol, Lee et al. (46) found that the increase of serum cholesterol was related to a better outcome in patients undergoing gastrointestinal surgery. Low serum cholesterol could cause reduced lipopolysaccharide binding and neutralization, a reduced number of circulating lymphocytes, limited tissue repair and regeneration, and dysfunction of the hypothalamic–pituitary–adrenal axis (47–49). To a certain extent, serum albumin and cholesterol can reflect the nutritional status of patients (45, 47). It is suggested that strengthening perioperative nutritional status may improve the short-term outcome of patients with colorectal cancer.

The present study has the following limitations: because this study was a retrospective cohort study, the authors had to rely on accurate records from the treating physicians. The incidence of rare postoperative complications is low and the confidence interval is large. As noted above, *p* values represent descriptive, exploratory summary measures of comparison only and do not represent confirmatory test results. Besides, this study measured visceral fat, subcutaneous fat, and skeletal muscle tissue area but not volume. This could lead to inaccurate estimates.

## Conclusion

Increased sarcobesity index is an independent risk factor for postoperative complications in patients with colorectal cancer, while visceral fat area is not. For female patients, smoking, operation time, and obesity index are independent risk factors for postoperative complications, while cholesterol is an independent protective factor. For male patients, serum albumin is an independent protective factor for postoperative complications. The number of dissected lymph nodes in sarcobesity patients was lower than that in general patients. Surgeons should therefore dissect lymph nodes more carefully in patients with sacrobesity, and be alert to the occurrence of postoperative complications in female patients with sacrobesity.

## Data availability statement

The original contributions presented in the study are included in the article/Supplementary material, further inquiries can be directed to the corresponding authors.

## Ethics statement

The studies involving human participants were reviewed and approved by Bioethics Committee of Beijing Friendship Hospital, Capital Medical University. The patients/participants provided their written informed consent to participate in this study.

## Author contributions

ZF contributed to conception and design of the study and performed the statistical analysis. KP and MT wrote the sections of the manuscript. XG, HL, and XY organized the database. YY and ZZ contributed to conception and design of the study. All authors contributed to the article and approved the submitted version.

## Funding

This work was supported by National Key Technologies R&D Program of China (Nos. 2015BAI13B09 and 2017YFC0110904).

## Conflict of interest

The authors declare that the research was conducted in the absence of any commercial or financial relationships that could be construed as a potential conflict of interest.

## Publisher’s note

All claims expressed in this article are solely those of the authors and do not necessarily represent those of their affiliated organizations, or those of the publisher, the editors and the reviewers. Any product that may be evaluated in this article, or claim that may be made by its manufacturer, is not guaranteed or endorsed by the publisher.
