# Supplementary material for: Sarcobesity, but not visceral fat, is an independent risk factor for complications after radical resection of colorectal cancer
Source: Front Nutr. 2023 May 16;10:1126127. doi: 10.3389/fnut.2023.1126127 (PMC10228740; doi:10.3389/fnut.2023.1126127)
Supplement: Supplementary file 2 [file Data_Sheet_1.docx]

**Supplemental Table 1** Multiple testing of surgical procedures between the stage I colorectal cancer group and the adenomatous polyp group

|  | χ2 | P |
| --- | --- | --- |
| Open | 0.321 | 0.571 |
| Laparoscope | |  |
| Open | 3.407 | 0.065 |
| Convert to open | |  |
| Laparoscope | 5.248 | 0.022 |
| Convert to open | |  |

Bonferroni correction was used to correct for multiple testing. The level of significance was tested at P<0.0167

**Supplemental Table 2** Number of risk factors in LASSO analysis. This number is consistent with those in Figure 2 and Supplementary Figures 1 and 3

| Number | Risk factors |
| --- | --- |
| 1 | VFA |
| 2 | SFA |
| 3 | TFA |
| 4 | V/S |
| 5 | Sacrobesity index |
| 6 | Age |
| 7 | Sex |
| 8 | BMI |
| 9 | Smoking |
| 10 | Diabetes mellitus |
| 11 | CCI |
| 12 | History of abdominal operation |
| 13 | Neoadjuvant therapy |
| 14 | Cancer type |
| 15 | Operative method |
| 16 | Stoma |
| 17 | Blood loss |
| 18 | Operative time |
| 19 | TG |
| 20 | HDL-C |
| 21 | Glucose |
| 22 | [Cholesterol](javascript:;) |
| 23 | CEA |
| 24 | CA199 |
| 25 | HGB |
| 26 | ALB |
| 27 | Nutrition status |
| 28 | ASA |
| 29 | Vascular invasion |
| 30 | TNM classification |
| 31 | Number of harvested lymph nodes |


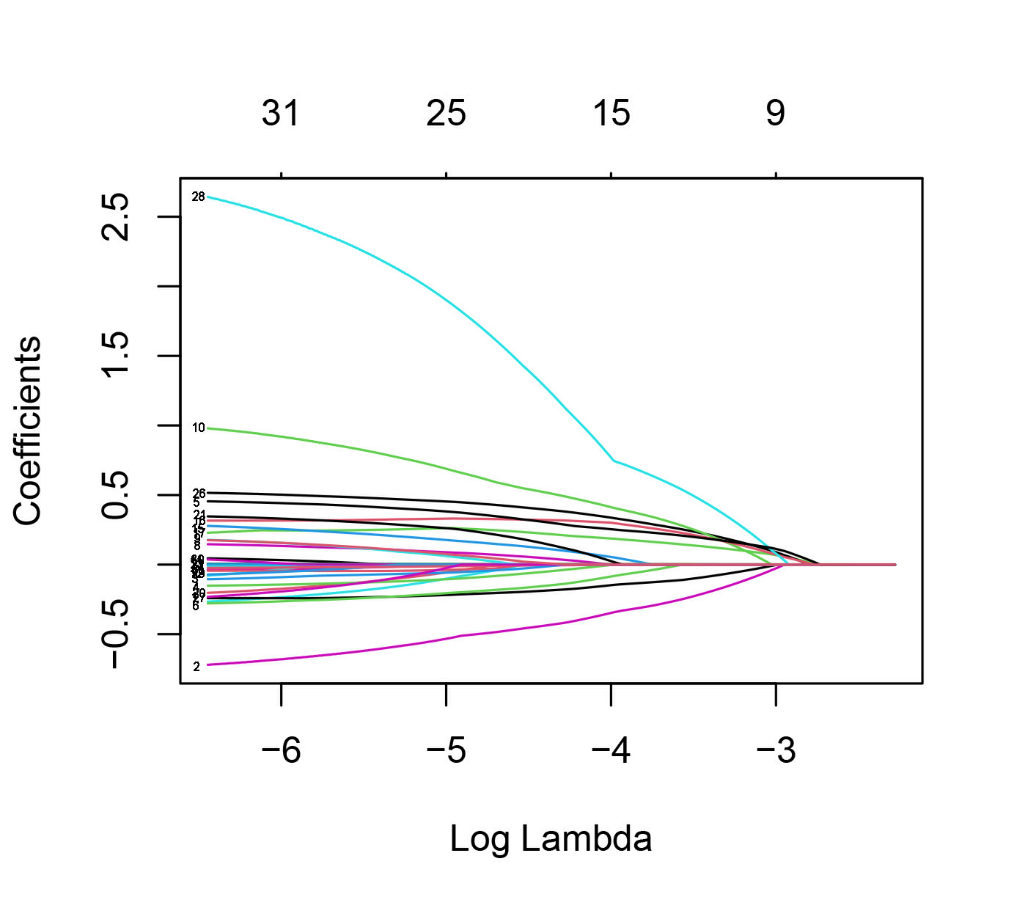


**Supplemental Figure 1** LASSO coefficient profiles of the 31 risk factors affecting the occurrence of postoperative total complications in male patients with colorectal cancer. The risk factors corresponding to the number of each curve are shown in Supplementary Table 2.

min


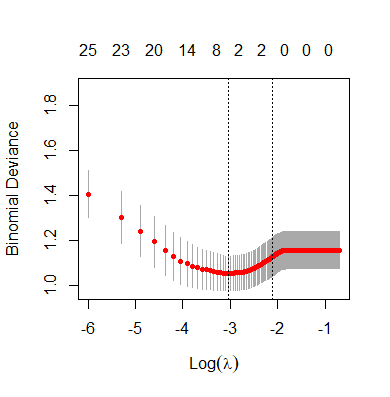


**Supplemental Figure 2** Five risk factors selected using LASSO Cox regression analysis. The two dotted vertical lines were drawn at the optimal scores by minimum criteria and 1-s.e. criteria.


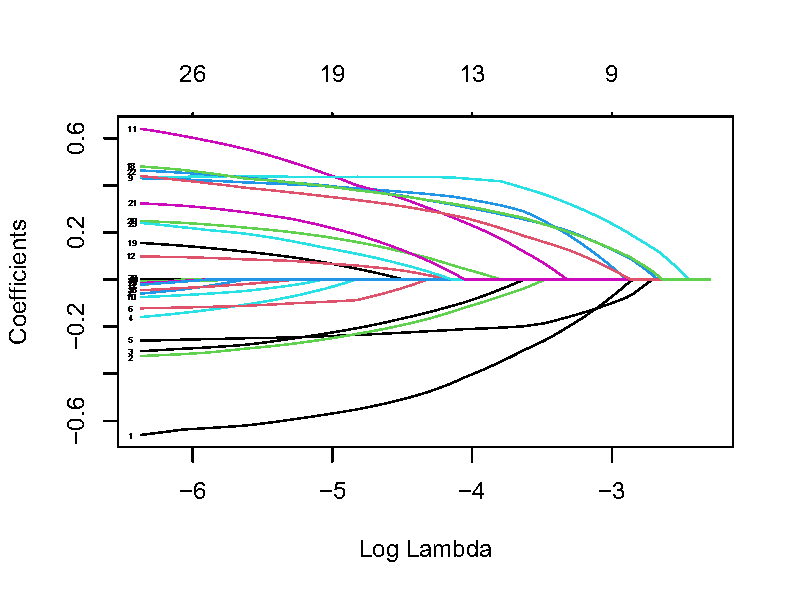


**Supplemental Figure 3** LASSO coefficient profiles of the 31 risk factors affecting the occurrence of postoperative total complications in female patients with colorectal cancer. The risk factors corresponding to the number of each curve are shown in Supplementary Table 2.


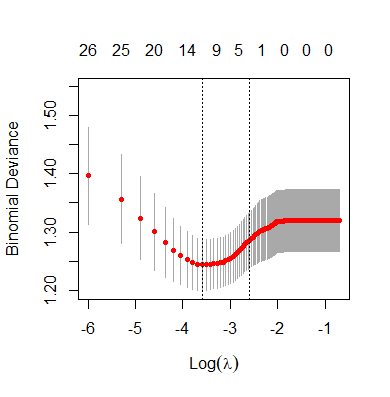


1-s.e.

min

**Supplemental Figure 4** Four risk factors selected using LASSO Cox regression analysis. The two dotted vertical lines were drawn at the optimal scores by minimum criteria and 1-s.e. criteria.
